# How to Improve Meat Quality and Welfare in Entire Male Pigs by Genetics

**DOI:** 10.3390/ani11030699

**Published:** 2021-03-05

**Authors:** Catherine Larzul

**Affiliations:** GenPhySE, Université de Toulouse, French National Institute for Agriculture, Food, and Environment INRAE, ENVT, 31326 Castanet-Tolosan, France; catherine.larzul@inrae.fr

**Keywords:** genetics, selection, boar taint, behavior, entire males

## Abstract

**Simple Summary:**

Successful breeding of entire male pigs needs a better understanding of factors driving meat quality and behavior traits as entire male pigs have lower meat quality, including an occasional strong defect known as boar taint, and more aggressive and sexual behavior. The review provides an update on how genetic factors affecting boar taint compounds and aggressive behavior in male pigs with emphasis on application in selection.

**Abstract:**

Giving up surgical castration is desirable to avoid pain during surgery but breeding entire males raises issues on meat quality, particularly on boar taint, and aggression. It has been known for decades that boar taint is directly related to sexual development in uncastrated male pigs. The proportion of tainted carcasses depends on many factors, including genetics. The selection of lines with a low risk of developing boar taint should be considered as the most desirable solution in the medium to long term. It has been evidenced that selection against boar taint is feasible, and has been set up in a balanced way in some pig populations to counterbalance potential unfavorable effects on reproductive performances. Selection against aggressive behaviors, though theoretically feasible, faces phenotyping challenges that compromise selection in practice. In the near future, new developments in modelization, automatic recording, and genomic data will help define breeding objectives to solve entire male meat quality and welfare issues.

## 1. Introduction

Public pressure to abandon castration has led stakeholders within the European Union to sign a voluntary declaration to end pig surgical castration. Giving up surgical castration will improve animal welfare owing to pain reduction during surgery, although aggression and mounting behavior may be detrimental to animal welfare in entire males during the finishing period. It will be beneficial for pig breeders as castration is an unpleasant task and non-castrated male pigs are more feed efficient, grow faster, and are leaner than castrates. It will also be beneficial for environment as entire male pigs excrete less nitrogen and phosphorus and have a lower contribution to global warming. Therefore, this change in pig breeding practice could largely improve environmental, social, and economic performance but is not without adverse consequences on meat quality. Regarding meat quality, this paper focuses on boar taint and will not consider the other meat quality traits that differ in entire males compared to castrates (fat quantity, fatty acid composition, tenderness, water holding capacity). Androstenone, a testicular steroid with a urine-like odor, and skatole, a product of the breakdown of tryptophan in the hind-gut, exhibiting a fecal odor, are the main compounds responsible for boar taint. Androstenone synthesis increases with sexual development. Fat skatole levels also tend to increase with age because its degradation is inhibited by androstenone and other testicular steroids.

Recently, in 2019, the EU health and food safety directorate-general published a guide establishing best practices for the production of male pigs, as well as the processing and the marketing of meat from uncastrated pigs or pigs vaccinated against boar taint [1]. This guide was established by a group of scientists on the basis of scientific publications and interviews of stakeholders that promote alternatives to piglet surgical castration. One part of the guide was dedicated to best practices to be set up at farm level to limit boar taint. Two statements from this report should be underlined. The first one is related to the importance of slaughter timing, as a mean to manage the risk of boar taint, based on either weight or age. The second statement is related to housing, generally associated with improved welfare, however it is not clear to what extent it impacts boar taint prevalence.

Best practices for boars include flooring and housing, early detection of pigs with boar taint, scheduling of slaughter, and transport to the slaughterhouse. Reductions in stress are associated with a reduction in boar taint as a consequence of less aggressive behavior and fewer fights between animals. Keeping pigs clean with fully or partially slatted floor has also been associated with reductions in boar taint. Sex separation is promoted though its effect on boar taint is not fully established. Avoiding mixing events limits stress, wet feeding, and competition avoidance at feeding decrease skatole levels. Age is generally considered to be a more effective measure on boar taint risk than weight, but weight cut-offs appear to be common industry practice even though maximum weight is not clearly specified and might depend on other factors, e.g., genetic type. There is no single threshold in age or weight that can guarantee the absence of boar taint. Finally keeping pigs clean and comfortable during transport is important for welfare, although it remains unclear whether this can also help reduce boar taint [1].

Management at the farm level will be efficient to limit skatole levels, especially via innovative feeding strategies, whereas genetic selection will decrease both skatole and androstenone levels.

## 2. Genetics for Better Quality in Entire Males

### 2.1. Breed Effect

Engesser [2] provided a thorough review of differences between pig breeds with view to boar taint and androstenone. One of the conclusions of the review pointed out the contradictions between studies, and that it would be difficult to classify completely breeds according to their boar taint risk. Additionally, no breed could claim an absence of boar taint risk. Overall, Hampshire and Duroc breeds present higher levels of androstenone. Differences between Landrace and Yorkshire or Large White are inconsistent, as Landrace may have lower, similar, or higher androstenone level. A recent study comparing German Landrace and Large White, raised under the same conditions, recorded higher average levels of androstenone and skatole in the Landrace breed [3]. Part of the difference could be attributed to different growth thus different weights at measurement. When compared under the same condition, around 100 kg body weight, Duroc pigs have a higher level of androstenone and a lower level of skatole than Landrace pigs [4,5,6,7,8]. The same genetic crossbreed can have different level of androstenone and skatole when provided by different breeding organization, as illustrated by [9].

**Figure 1:**
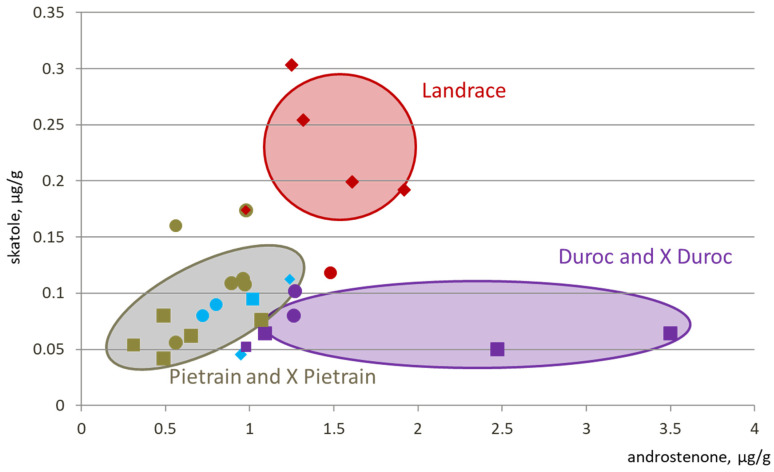
Levels of androstenone and skatole in different breeds and crossbred populations [5,7,10,14,15]. Shape for the lines: square = paternal line, diamond = maternal line, circle = crossbred; color for the breed: beige = Pietrain, red = Landrace, blue = Yorkshire or Large white; purple = Duroc; for crossbred, the color of the paternal line.

The Figure 1 illustrates the average androstenone and skatole levels in different purebred and crossbred populations. Data were taken as published, even though differences were not only due to genetic type. They could have been induced by different origins, farming systems, measurement methods, and so on. Pietrain breeds as well as Pietrain crossbred have a lower risk of boar taint than other breeds, Landrace breeds have higher level of skatole but not related to a higher level of androstenone, and Duroc lines tend to have high levels of androstenone with a low level of skatole. In general, sire lines have lower boar taint risk than dam lines [10]. The differences are explained by the different breeding objectives in these lines. Maternal lines are selected on reproduction characters and numeric productivity and paternal lines are mainly selected on growth rate, feed efficiency, and leanness. Because of genetic selection process, differences between lines may evolve over time. For example, Bonneau et al. [11] observed higher levels of androstenone in Pietrain carcasses than in Belgian Landrace carcasses. Following Sellier [12], and partially Frieden et al. [13], Pietrain boars would be less desirable for entire male production. Conversely, all recent studies promote the use of Pietrain sire lines to decrease boar taint risk. However, there is still a lack of information on a large number of breeds, especially local breeds. The level of boar taint risk will remain unknown in these breeds until a significant number of androstenone and skatole measurements are performed.

### 2.2. Heritability and Genetic Correlations for Boar Taint Related Traits

Genetic determinism of boar taint has been reviewed several times [2,12,16,17].

The heritability of boar taint, estimated as an overall trait, has a low (h^2^ = 0.13, [18]) to high (h^2^ = 0.91, [19]) value, with a mean heritability of h^2^ = 0.54 [12]. Because of the difficulties to collect a large amount of data, only a few studies provide precise heritability values for boar taint or boar taint risk, measured by sensory evaluation.

Windig et al. [10] and Mathur et al. [14] estimated low heritability values (h^2^ = 0.11 to 0.19, merging different breeds and crossbreeds, h^2^ = 0.14 in Pietrain breed) for boar taint assessed by human nose scoring on a large number of carcasses in the slaughterhouse. In both studies, human nose was performed by experts selected according to sensitivity to boar taint compounds.

Studies on genetic determinism of boar taint focused on heritability of boar taint compounds, androstenone and skatole, and the genetic relationship between the two compounds. In the bibliographical review from [20], the heritability of androstenone content was estimated to 0.54 on average, ranging from 0.25 to 0.88. Heritability of skatole content is lower, ranging between 0.19 and 0.54 [16]. Including recent studies, the published heritability values for fat androstenone and fat skatole contents, as well as the genetic correlations between the two compounds, are reported in Table 1. Most of the genetic parameters were estimated in Landrace, Large White, Duroc, and Pietrain breeds. Most of the estimates range between 0.4 and 0.6 for androstenone and between 0.3 and 0.6 for skatole. Willeke et al. [21] published realized heritabilities from a selection experiment with two divergent lines selected for high or low backfat androstenone, with similar heritability values in both lines (h^2^ = 0.58 and h^2^ = 0.51, respectively).

Use of genomic information provided new insights in genetic determinism of boar taint compounds. Tusell et al. [22] estimated the part of dominance variance for androstenone and skatole levels in purebred Pietrain and Pietrain × Large White crossbred. In their model, the genetic parameters were estimated using genomic information, following a modelization where purebred and crossbred performances were considered as two different traits. In the crossbred population only the part of variance from the sire line was estimated [22]. When dominance effect is included in the model, heritability values tend to be lower [15,23] (Table 1). The dominance part was low in purebred (h^2^_D_ = 0.06) but was significant in crossbred (h^2^_D_ = 0.13). The heritability values estimated with genomic information were in the medium range for androstenone in all studies (h^2^ = 0.17 to 0.40 [24], h^2^ = 0.28 to 0.31 [25], h^2^ = 0.46 [26]) but heritability values for skatole tended to be lower (h^2^ = 0.14 to 0.38 [24], h^2^ = 0.07 to 0.1 [25], h^2^ = 0.26 [26]). The low genomic heritability values for skatole could be due to the genetic architecture of the trait, with the segregation of a quantitative trait locus (QTL) having significant effect on the skatole level [25]. The frequency of the different alleles might differ from one population to another, leading to different genomic heritability values. Dugué et al. [15] estimated a high genetic correlation (rg = 0.74) between fat androstenone measured in Purebred Pietrain and related Pietrain × Large White crossbred raised under the same conditions. This correlation tends to demonstrate that genetic determinism might differ between two genotypes as the correlation differ from 1.

The genetic influence on other individuals from the same group was also studied. Duijvesteijn et al. [27] estimated the variance due to indirect genetic effects for androstenone level in a Duroc population. Indirect genetic effects can be described as the genetic influence of an individual on its pen mates. The model with social effects performed better and provided a higher total heritability (T^2^ = 0.75, which includes both additive variance and indirect genetic variance) than the basic model (h^2^ = 0.63 which includes only additive variance). The positive (rg = 0.24, but not significantly different from 0) correlation between direct genetic effect and indirect genetic effects tends to support the hypothesis that individuals with high androstenone levels induce higher androstenone levels in their pen mates [27].

The heritability values for androstenone were mostly estimated in backfat but one study provided heritability values for androstenone measured either in plasma or in backfat in two breeds [7]. For Landrace, the heritability values were very similar (h^2^ = 0.47 ± 0.08 and h^2^ = 0.49 ± 0.08, respectively) with a very high genetic correlation between both traits (rg = 0.98 ± 0.03). For Duroc, the heritability values were quite similar (h^2^ = 0.56 ± 0.11 and 0.67 ± 0.10, respectively) with also a very high genetic correlation between both traits (rg = 0.91 ± 0.05). The genetic correlations between plasma estradiol and backfat androstenone is very high, ranging from 0.8 to nearly 1 [7,15,23], suggesting that estradiol can be efficiently used to select against androstenone.

The genetic correlation between backfat androstenone and skatole contents is positive (around 0.4) with values ranging between 0.2 and 0.6 (Table 1). Windig et al. [10] estimated genetic correlations between human nose score for boar taint and androstenone or skatole level. The genetic correlations were higher with skatole (rg = 0.90) than with androstenone (rg = 0.65), but in some cases genetic correlations were estimated with large standard errors (up to 0.5). It is unclear whether the different correlations are due to higher difficulties to detect androstenone or to the characteristics of the populations studied. Genetic correlations were also estimated between androstenone and skatole levels measured at two different ages, the first measurement being made on live animals and the second one on fat sampled from the carcass at the slaughterhouse. The genetic correlation was nearly 1 (rg = 0.97, [28]) between 100 kg and 117 kg whereas the genetic correlation was much lower when the delay between the two measurements was increased (rg = 0.67 between 110 kg and 160 kg, [29]). In both cases, the heritability value was higher for the carcass measurement (h^2^ = 0.49 and 0.55 respectively [28]; h^2^ = 0.39 and 0.58 respectively [29]). By the way, in those studies, it remains difficult to disentangle the effect of age or weight from sampling conditions.

### 2.3. Genetic Relationships between Boar Taint and Traits of Economic Interest

In their review, Parois et al. [17] reported that a selection against androstenone does not induce adverse effect on growth rate and carcass composition but it may indirectly alter some maternal traits [22,32].

The genetic relationships between boar taint and production or meat quality traits are considered as either favorable or very low. In fact, there are few published genetic correlations between boar taint traits and production traits in finishers such as growth rate, feed efficiency, carcass composition, or meat quality traits. In their study dedicated to elaborate strategies to select against boar taint, Haberland et al. [33] reported no genetic correlations between androstenone or skatole and average daily gain, low correlations with feed efficiency (rg = 0.13 for androstenone and rg = 0.14 for skatole) and negative correlations with leanness traits (around rg = −0.2 for androstenone and rg = −0.15 for skatole). The same authors mentioned a low correlation between androstenone or skatole and intramuscular fat (rg = 0.19 and rg = −0.04, respectively). Genetic correlations between androstenone and growth rate, either estimated by average daily gain or body weight at fixed age are negative (rg from −0.23 to −0.04 [3,10,15,28,29,31]) or slightly positive (rg = 0.04, [15,28,29]). Except for the moderate genetic correlations estimated between skatole and growth rate (rg = −0.51 at 160 days or age and rg = 0.34 at 220 days of age [29]), the correlations between these two traits are low (rg = −0.04 to −0.10 [10,31]). The genetic correlations between carcass leanness, that can be measured by backfat thickness, loin muscle area, or carcass meat percentage, are consistently moderately negative with androstenone level (rg = −0.10 [10], rg = −0.15 [15], rg = −0.26 [29], rg = −0.22, rg = −0.23 [33] and rg = −0.18 [31]) and skatole level (rg = −0.10 [10], rg = −0.24 [29], rg = −0.16 rg = −0.12 [33] and rg = −0.20 [31]). Dugué et al. [15] estimated a medium genetic correlation between androstenone and feed conversion ratio (rg = 0.47), ultimate pH (rg = −0.20 and rg = −0.40 depending on muscle) and a null correlation between androstenone and intramuscular fat (rg = −0.04). Strathe et al. [31] estimated low genetic correlations between feed conversion ratio and androstenone rg = (−0.04) or skatole (rg = 0.18). But for few exceptions that can be related to population or the age at the time of measurement ([29] for example), the genetic correlations between boar taint compounds and finisher production traits should be considered as either low or favorable when higher growth rage, better feed efficiency, higher carcass leanness, or high ultimate pH are expected. Genetic correlations between production traits and human nose score could be considered as null [10].

Other meat quality traits might be of interest regarding entire males, such as tenderness or fat quality. For example, Mörlein and Tholen [34] observed higher concentrations in polyunsaturated fatty acids in pigs with very low concentrations of boar taint compounds compared to those with high levels. Genetic determinism of meat quality traits has been largely studied (reviewed in [20]) but to the best of our knowledge, the genetic relationships between boar taint and organoleptic traits have not yet been sufficiently evaluated.

Because of the strong relationship between gonadal synthesis of androstenone and other steroids [35], selection against boar taint could interfere with reproduction performances in both males and females. However, results from selection experiments or genetic parameter estimations have been inconsistent. As reported by Engesser [2], Jonsson and Wismer-Pedersen [36] found no significant antagonistic effect between sex odor intensity with boar’s libido or with litter size at birth. In their selection experiment on androstenone, Willeke et al. [21] described a delay for the first heat in gilts associated with low androstenone levels. Sellier and Bonneau [32] also observed a delay in puberty in gilts as a response to selection against boar taint. Lervik et al. [37] observed no effect of the estimated breeding value for androstenone on the morphology of the testes suggesting that breeding against androstenone should not influence the testicular development during the pubertal process.

Genetic correlations estimated between boar taint compounds and male or female reproductive traits suggest that that selection against boar taint would have no large effects on reproductive traits, but authors of the different studies tend to be cautious in their conclusion because these parameters are difficult to estimate with high precision. The genetic correlations estimated between androstenone or skatole and bulbo-urethral glands development, as a predictor of sexual maturity, were moderate and positive (rg = 0.38 to 0.57 [5]; rg = 0.34 to 0.68 for androstenone only, [28]). Brinke et al. [3] reported negative correlations between sperm volume with androstenone (rg = −0.18 and rg = −0.25), null correlations (rg = 0.04 to 0.08) between androstenone and sperm density, skatole, and sperm volume or skatole and sperm density, except for a moderate positive genetic correlation between these two traits in the German Large White population (0.32). Regarding sexual development in females, Sellier et al. [28] estimated low or positive genetic correlations between androstenone and age at first estrus (rg = −0.08 and rg = 0.22). According to Mathur et al. [14] and Brinke et al. [3], genetic correlations with age at first insemination were low value for androstenone (rg = −0.10 to 0.04) and highly contrasted for skatole (rg = −0.34 to 0.36, depending on the breed). Genetic correlations between androstenone or skatole and litter size tend also to be contrasted depending on the breed (rg = −0.25 to 0.31 [3,14,31]). Applying a more complex modelization, Strathe et al. [38] estimated favorable genetic correlations between boar taint and eight traits indicators of reproductive performance for males (sperm characteristics) and females (litter size and piglet survival). As underlined by the authors, the correlations were estimated with large standard errors, partly because of the lack of common observations on boar taint and fertility traits, which is generally the stumbling block of studies reporting genetic correlations between boar taint and reproductive traits (especially in females).

### 2.4. Selection against Boar Taint

Considering the genetic determinism of boar taint or boar taint compounds, selection for better meat quality has been under consideration for several decades. There is a consensus that selection against boar taint is possible and would be beneficial to the entire male pig production. Given the low genetic correlations with growth performance, selection against boar taint in sire lines should have little impact on genetic progress on growth rate and carcass composition. Provided that the low impact of selection against boar taint compounds on maternal traits is confirmed, the cost of performing such a selection in dam lines should also be bearable.

The first attempt to prove that selection against boar taint would be efficient was experimentally carried out by Jonsson and Andresen [30] in a Landrace population. They successfully decreased backfat androstenone and boar taint odor, using a selection that combined androstenone level in backfat and a panel evaluation of boar taint. Willeke et al. [21] experimentally confirmed that selecting on androstenone level in backfat would be efficient, but also pointed out that selection to decrease androstenone level might reach a plateau meaning that decreasing androstenone level down to 0 would be impossible. This observation still remains to be confirmed. A third selection experiment was carried out in Landrace × Large White population by Sellier et al. [28]. The aim of the study was to demonstrate the feasibility of a selection against boar taint without affecting sexual maturation, the constraint being on the size of the bulbo-urethral glands. They provided evidence that selection against boar taint with constraint could be realized and proved that the estimation of adequate genetic parameters was of primary importance. Up to now, direct selection against skatole has not been experimentally demonstrated.

Several authors compared the effect of different breeding strategies against boar taint based on published genetic parameters. One of the major concerns regarding selection against boar taint was potential unfavorable effects on production and reproduction traits. Based on very simple assumptions, i.e., ignoring negative changes in reproductive traits, Ducro-Steverink [39] estimated that the incidence of boar taint could be reduced from 30% to 10% in less than 5 years. Frieden et al. [13] compared two different breeding strategies for sire lines and dam lines, assuming that androstenone can be measured on live candidates from backfat biopsy samples. Because sire and dam lines differ considerably on their selection objectives, different economic weights were provided for both lines, with more emphasis on female reproductive traits in dam lines and more emphasis on growth, feed efficiency, and leanness in sire lines. The conclusion of the study was that inclusion of the androstenone level to achieve a reduction of boar taint would result in significantly less progress in reproductive performances in dam lines, and considerable progress in meatiness would be sacrificed in the sire line. Up to six generations would be necessary to reduce the frequency of “risky” boars from 20% to 5%. Considering their own parameter estimates, Mathur et al. [14] estimated that a selection on the reproduction traits only would increase the percentage of the carcasses with boar taint from 4 % to 7.3% within five generations. A long-term balanced selection, combining boar taint and reproduction traits, would eventually (19 generations) reduce percentage of tainted carcasses from 4% to below 0.1%, with increased economic gain (but only in markets with intact male pigs). Haberland et al. [33] simulated different scenarios for selection against boar taint in a sire line, considering simultaneously the implementation of genomic selection and economic consequences. The authors concluded that the most efficient strategy would be to include a direct measurement of androstenone in candidate boars based on a fat biopsy sample combined with genomic selection.

All the scenarios were based on a selection in purebred where it is expected to improve crossbred pigs. It is understood that this selection is efficient when genetic correlation is high between purebred and crossbred performances but it has been evidenced that the genetic correlation is around 0.7 [15]. Under those circumstances, using crossbred performances for selection might prove more appropriate.

Different genomic prediction methodologies were compared by De Campos et al. [26] and Azevedo et al. [24] for selection against boar taint. Because the different methods performed differently on all the studied traits, it was difficult to promote one methodology over the others. As genomic selection implementation is extending in pig breeding, the single-step genomic evaluation [40] tends to predominate and other methodologies are not really under consideration for routine evaluation.

### 2.5. Selection against Boar Taint in Practice

Some breeding companies have included boar taint into the breeding objective of sire lines. Engessen [2] reported some details on the creation of three “low boar taint” sire lines: “db.7711,” “Inodorus 2.0” in Germany and “Nador” from Topigs-Norsvin. There was some research in Switzerland by Suisag for selecting against boar taint the Premo^®^ line but it was abandoned since then (personal communication). A study reported by Engessen [2] compared the “Inodorus” boar and “Nador” boar without detecting significantly different levels of boar taint compounds between the two lines. The results confirmed that selection was successful in reducing boar taint. Details on the different lines selected against boar taint can be found in the best practices guide [1]. Topigs-Norsvin has also succeeded in reducing boar taint instance across their various lines (Talent, Tempo and Top Pi). For example, in the Talent line, progeny with boar taint decreased from nearly 6% down to 2.5% between 2008 and 2017; Topigs-Norsvin estimates a 50% reduction in odorous carcasses in their populations within 10 years and indicates that the Nador line reduces the risk of boar taint to less than 1% in slaughtered pigs. Eventually, they implemented selection against boar taint in all their lines to secure the reduction of boar taint risk in the final product. In France, the breeding company Nucleus developed the “Ino” low boar taint Pietrain line. By generalizing the use of Ino boars, the percentage of tainted carcasses measured at slaughterhouse decreased from 4% to 2% between 2017 and 2018 [1]. Breeding company PIC developed a strategy to provide low-taint sperm but PIC has indicated that there have been some issues with pigs bred for low taint. Pigs bred with low-taint sperm have bellies of reduced size and quality and low-taint sperm may negatively affect sow productivity [1]. The details of selection strategies established by breeding companies are very partially known. The Nador selection program is based on the human nose score, the analysis of boar taint compounds in fat samples and DNA analysis. Breeding company Nucleus has implemented selection against boar taint based on a prediction of boar taint risk estimated from a blood sample.

Selection objectives are generally based on a large number of traits, differing from one line to another. As mentioned previously, sire lines emphasize growth rate, feed efficiency, leanness, and meat quality whereas dam lines emphasize reproductive traits such as age at puberty, litter size, piglet viability, maternal abilities, and fertility. Thus, it is of primal importance to know the genetic relationships between boar-taint-related traits and traits of interest implemented in breeding objectives. On the contrary, not taking into account boar taint in the selection objectives might lead to an increase of boar taint compounds over time. For example, Andersen-Ranberg et al. [8] estimated a genetic trend between 2002 and 2016 for androstenone and skatole levels in a Landrace and a Duroc populations. They observed no evolution in the Landrace whereas there was a clear increase but only for androstenone (0.16 µg/g/year) in the Duroc population. The origin of these contrasted genetic trends were not unraveled but they could be explained either by different genetic correlations with traits under selection or different selection objectives in the two lines.

## 3. Genetics for Better Entire Male Welfare

The other issue for entire male production is to overcome aggressive behaviors and sexual or mounting behaviors observed in entire males [41]. Behavior traits are known to have a genetic basis and heritability values have been estimated for several of behavior related traits. However, selecting for, or against, specific behavioral traits is not straightforward [42]. For example, agonistic behaviors in pigs are useful to establish the hierarchy in the group and in general they are limited when the group is kept unchanged. Then, selecting against agonistic behavior should be focused on limiting the damaging consequences of excessive aggressiveness, and not directly against agonistic behavior. The selection against sexual behaviors, or mounting behaviors, should also be carefully evaluated as mounting behavior is part of the pigs’ normal behavioral repertoire [43] primarily related to the expression of libido [44]. Selecting against this behavior might eventually impair reproductive ability.

If entire males are expected to show a higher degree of aggressiveness, all sex categories express agonistic behaviors. These behaviors are exacerbated after mixing events (sometimes causing deep injuries) which are common practices in pig breeding from birth (with adoption) to adulthood (sows kept in group during pregnancy periods). The entire males’ aggressive behaviors are believed to be caused by the changed hormonal status at puberty, with an increased level of testicular steroids in entire males [45]. The high genetic relationship between number of skin lesions at the end of the finishing period and testosterone level (rg = 0.82) largely supports this hypothesis [23]. The skin properties should also be taken into account to deepen our understanding of the genetic determinism of skin lesions.

Genetic parameters of aggressive behaviors have been reviewed on numerous occasions (for some recent reviews [42,46,47,48]) and genetic models to provide useful strategies to select against damaging behaviors were recently detailed by Canario et al. [49], but previous literature rarely focused on entire male behavior specific issue.

### 3.1. Breed Comparison

There are fewer comparisons between breeds for aggressive behavior but differences on aggressive behavior-related traits were observed between breeds. Large White pigs seemed to be more aggressive than Berckshire [50]. Duroc pigs are more aggressive than Landrace pigs [51] or Yorkshire pigs [52]. No significant differences were observed between Large White and Landrace pigs [53] or between Yorkshire and Yorkshire x Landrace pigs [54] for aggressive behavior traits or skin lesions records. These studies were performed on females and barrows. Prunier et al. [55] observed a higher number of skin lesions on Pietrain × Large White crossbred boars than on Pietrain boars, but differences were limited between the two genotypes at the end of the finishing period when observed in stable groups. Dugue et al. [15] confirmed these differences on a higher number of individuals and hypothesized that due to the sensitivity to stress with the occurrence of the *n* allele at the RYR1 gene, Pietrain may have been indirectly selected for lower aggressiveness. Desire et al. [56] studied females and castrated from seven different commercial lines and mentioned a significant effect of the line on skin lesions recorded after post-weaning without detailing the differences between the lines. Chu et al. [57] observed a very low level of aggressive behavior in Meishan females, with nearly no observable skin lesions, compared to Large White females pigs, both at a young age and after mixing.

### 3.2. Genetic Parameters for Skin Lesions and Aggressive Behavior and Selection Strategies

Genetic determinism studies of aggressive behaviors or mounting behaviors need a large amount of data that are generally difficult to record, costly, and time consuming. As repeatedly mentioned [47], several traits have been proposed as easily measured indicators of involvement in aggressive behavior, but the most reliable and practicable approach for large sample sizes is the counting of skin lesions resulting from regrouping.

The heritability for aggressive behavior at mixing recorded on different sex categories including males, castrates, or females at different physiological stages, varies between 0 and 0.44 (for review, Canario et al., submitted). In studies with individuals observed on a short period where bullying events could be quantified, heritability was estimated very low [54] or moderate (h^2^ = 0.37 [58]) and reciprocated fighting and the delivery of non-reciprocated bullying have moderate heritabilities in pigs (h^2^ = 0.17–0.43 [58]).

The benefits of reducing aggression at mixing at young age may not be adequate to limit skin lesions in stable groups [59]. Though positive, the genetic correlations between lesions at mixing and lesions in stabilized group are only moderate (rg = 0.28 to 0.50, [54], but up to 0.76, [56]) and might depend on sexual type or environmental factors. In the study described by Parois et al. [23], skin lesions were counted on Pietrain and Pietrain × Large White entire male piglets at three periods. On live piglets, lesions were counted 48 h after entering the fattening pen (mixing event), the day before the first departure to slaughterhouse (stable group) and on carcasses. The heritability values for the three traits were h^2^ = 0.25, h^2^ = 0.14 and h^2^ = 0.30 in purebred and h^2^ = 0.25, h^2^ = 0.20 and h^2^ = 0.36 in crossbred. The heritability values were higher when measured on carcass, related to a higher skin lesion number. The genetic correlations between the three periods are high in purebred (rg = 0.73 to 0.74) but unexpectedly very low in crossbred ((rg = −0.05 to 0.23). Skin lesions measured on carcass can be also a trait of interest because when animals fight at slaughterhouse, they consume glycogen and meat pH increases. Though skin lesions are highly related to sexual maturation in stable groups of entire males [15], the genetic correlations between skin lesions and androstenone level is only moderate (around 0.2, [15]).

Skin lesions appear as a quite simple trait to record, either on live animals or on carcass, after mixing events or in stable group, but it has not been considered in breeding schemes to decrease the entire male aggressiveness assumedly because recording remains too time consuming.

Canario et al. [49] detailed two alternatives that might guide selection against aggressive behaviors in entire males. The first one is to select on social (indirect) genetic effects. For example, selecting individual with high social genetic breeding values for growth rate would reduce expression of aggression in stable groups [60,61,62]. The estimation of social genetic effects for growth rate might differ between sexes as shown by Nielsen et al. [63], and then it would be important to consider the social genetic effects as different traits between females and entire males. Entire males showed a higher variance and, contrary to entire males, gilts showed genetic effects of competition. Additionally, to assess social genetic effects properly, grouping animals cannot be made at random and choosing pen mates according to family structure is of primal importance.

Recently, Angarita et al. [64] developed an alternate model to take into account the observational data in estimating social genetic effects on skin lesions recorded on gilts and barrows. They concluded that selection incorporating social genetic effects may greatly reduce the consequences of damaging aggressive behavior after mixing pigs in new social groups but it would be necessary to confirm the relevance of this approach in stable groups of entire males.

A second option described by Canario et al. [49] is to better understand the relationships between individuals at the group level and not only by pair of individuals as described in dyadic recording of aggressions. The position of each individual in the group can be described by centrality parameters estimated by social network analysis [65]. An example of such an analysis on pig behavior can be found in Foister et al. [66]. The information needed to build the edges (i.e., links between two individuals of the network) remains to be developed but it has been suggested to use automatic recorded data. Using data from automatic feeding device, Larzul et al. [67] estimated a high genetic correlation between skin lesions measured on entire male pig carcass and betweenness centrality (rg = −0.81) or closeness centrality (rg = −0.26). These two indirect approaches have the advantage to use available data recorded on a routine base in some nucleus population. Their efficiency in reducing aggressive behavior in entire males need further investigation.

Genetic determinism of mounting behaviors in entire male finishers remains to be studied.

## 4. Perspectives

One of the major challenges in breeding entire males is access to relevant data. Selecting for better meat quality and adequate behaviors is clearly impaired by the limited implementation of cheap and rapid measurement methods, particularly on live animals.

### 4.1. New Phenotyping

Regarding meat quality, new developments are available or will be in the next future due to the need to sort tainted carcasses at slaughter plants. The most promising device for boar taint is based on LDTD-MS/MS (laser diode thermal desorption-tandem mass spectrometry) technology but electrochemical biosensor system or spectroscopy technology are also under development. When available, genetic studies can be carried out on these new traits to evaluate their potential for selecting better meat quality in entire males.

Regarding behavior, the development of automated recording is abounding. An extensive review was recently published for machine vision technology by Wurtz et al. [68] or combination of technologies [69]. For example, Nasirahmadi et al. [70] and Li et al. [71] developed an approach to detect mounting behaviors in pigs by automated image analysis whereas Oczak et al. [72] developed image analysis to automatically detect fighting. Specific developments are also provided to be able to track individuals [68] which is of high importance for genetic studies.

There are also various projects on automated detection of behaviors or harmful social interactions based on non-visual technologies that offer new perspectives in animal breeding (UHF RFID, [73]; infrared thermography, [74]; accelerometers [75]). All these technologies are promising and should be investigated in the near future for genetic behavioral studies.

### 4.2. Genomic Information

The use of SNP arrays to carry out genomic evaluation is spreading in pig breeding to improve prediction accuracy and increase genetic progress per generation. Several studies have provided information either on QTL or candidate genes with an effect on boar taint related traits ([76] for a recent review). Up to now, no clear mutations have been evidenced that would be of direct interest to select against boar taint, except those resulting from genome editing [77] for a recent review). On the contrary, a very large number of QTLs or candidate genes have been published for meat quality traits (https://www.animalgenome.org/QTLdb). A meta-analysis performed on this large number of QTL was an efficient methodology to consolidate results and propose valid information to assist selection [78]. Even though causal mutations are unknown, genomic markers can offer useful strategies in local breeds in which boar taint risk is poorly known. It is possible to estimate the frequencies of the alleles of genes known to be related to different skatole or androstenone level in local breeds [79] that can provide opportunities to decrease boar taint by selecting favorable alleles.

As far as aggressive behavior is concerned, whole genome studies are scarce in pigs [80,81] as well as exploration of candidate genes or genomic markers [82,83,84,85,86]. Wurtz et al. [81] evidenced only one region on chromosome 11 for skin lesions that need to be confirmed. A better understanding of genetic determinism of aggressive behaviors, but also positive behaviors, should be accessible with the development of new phenotyping as genomic data are more and more affordable.

## 5. Conclusions

To support the transition to the abandonment of castration and the rearing of entire males in pig production, the genetic selection brings medium to long-term solutions to control the problems of boar taint and aggressive behavior. Some breeds are identified as low-taint risk and can be used for crossbred pigs. Because of the knowledge developed over many years on the genetic determinism of skatole and especially androstenone, breeding organizations have recently developed selection strategies to propose improved lines for the breeding of entire males. However, research has mainly focus on conventional breeds. Local breeds are still little studied and promoting the abandonment of castration in production systems using these breeds is an uncertain step.

In order to generalize the selection against boar taint and improve the overall quality of meat from entire males, further studies are needed to evaluate the genetic relationships between boar taint and organoleptic or fat quality traits, as well as relationships with reproductive traits. As far as aggressive behavior is concerned, though theoretically feasible, implementation of selection for more friendly individuals is not under consideration. However, in the near future, genetic studies and selection will highly benefit from the development of new technologies, especially regarding automatic measurements of boar taint compounds at the slaughterhouse or individual behaviors on farm. Combining genomic information with automatic recording should provide fascinating opportunities to develop new breeding strategies and face challenges related to entire male pig production.

## Figures and Tables

**Table 1:** Genetic parameters for androstenone and skatole levels measured in backfat.

Breed/Population	Heritability		Genetic Correlation	Reference
	Androstenone	Skatole		
French Large White × Landrace	0.49; 0.55			[28]
French Pietrain	0.63	0.37	0.61	[22]
French Pietrain × Large White	0.70	0.24	0.36	
French Pietrain	0.38 *	0.14 *		[15]
French Pietrain × Large White	0.54 *	0.27 *		
French Pietrain	0.57			[23]
French Pietrain × Large White	0.71			
Italian Large White (160 days)	0.39	0.60	0.30	[29]
Italian Large White (220 days)	0.58	0.60	0.56	
German Landrace	0.56			[20]
German Landrace	0.50	0.52	0.29	[3]
German Large White	0.39	0.32	0.41	
Norwegian Landrace	0.54	0.55	0.36	[5]
Norwegian Duroc	0.56	0.23	0.62	
Norwegian Landrace	0.49	0.41	0.32	[7]
Norwegian Duroc	0.67	0.37	0.33	
Norwegian Landrace	0.45	0.53	0.32	[8]
Norwegian Duroc	0.50	0.33	0.42	
Dutch synthetic	0.75	0.53	0.22	From [2]
Dutch Duroc	0.46	0.26	0.24	[26]
Dutch Duroc	0.63			[27]
Multibreed	0.69	0.53		From [2]
Multibreed	0.64	0.26		From [2]
Multibreed	0.54	0.41	0.37	[10]
Dutch Pietrain	0.72	0.69		[14]
Dutch Landrace and Yorkshire	0.73	0.82		
Swiss Large White	0.45	0.50	0.11	From [2]
Danish Landrace	0.25; 0.54			[30]
Danish Landrace	0.47–0.49	0.54–0.56		From [2]
Danish Landrace	0.59	0.33	0.35	[31]
Danish Landrace	0.27–0.31	0.06–0.11		[25]
Danish Landrace	0.52	0.28	0.38	From [2]
Landrace		0.27		From [2]
Large White		0.19		
Duroc		0.19		
Hampshire		0.21		
Pietrain	0.61	0.50		From [2]

* Estimated with a dominance effect in the model.

## Data Availability

The study did not report any data.
